# Hierarchically structured nanowires on and nanosticks in ZnO microtubes

**DOI:** 10.1038/srep15128

**Published:** 2015-10-12

**Authors:** C. M. Rivaldo-Gómez, G. A. Cabrera-Pasca, A. Zúñiga, A. W. Carbonari, J. A. Souza

**Affiliations:** 1 Universidade Federal do ABC, Santo André– São Paulo 09210-580, Brazil; 2 Instituto de Pesquisas Energéticas e Nucleares, Universidade de São Paulo, 05508-000 São Paulo, Brazil

## Abstract

We report both coaxial core-shell structured microwires and ZnO microtubes with growth of nanosticks in the inner and nanowires on the outer surface as a novel hierarchical micro/nanoarchitecture. First, a core-shell structure is obtained—the core is formed by metallic Zn and the semiconducting shell is comprised by a thin oxide layer covered with a high density of nanowires. Such Zn/ZnO core-shell array showed magnetoresistance effect. It is suggested that magnetic moments in the nanostructured shell superimposes to the external magnetic field enhancing the MR effect. Second, microtubes decorated with nanowires on the external surface are obtained. In an intermediate stage, a hierarchical morphology comprised of discrete nanosticks in the inner surface of the microtube has been found. Hyperfine interaction measurements disclosed the presence of confined metallic Zn regions at the interface between linked ZnO grains forming a chain and a ZnO thicker layer. Surprisingly, the metallic clusters form highly textured thin flat regions oriented parallel to the surface of the microtube as revealed by the electrical field gradient direction. The driving force to grow the internal nanosticks has been ascribed to stress-induced migration of Zn ions due to compressive stress caused by the presence of these confined regions.

Semiconductor coaxial and tubular micro/nanostructures with hierarchical morphologies have been investigated with great interest because both fundamental science and potential applications in diverse technological fields^1^,^2^,^3^,^4^. Micro/nanowire materials are fascinating systems for the investigation of electrical transport, optical, magnetic, and mechanical properties^5^,^6^, whereas semiconducting and ferromagnetic combined functionalities are important for technological application in spintronic devices. For example, ZnO nanostructured materials are suggested to exhibit ferromagnetic-like properties, which have been attributed to the presence of intrinsic defect/strain^7^,^8^. Taking advantage of magnetic and electric properties, such as spin dependent electron transport and phase transition from insulator to metallic behavior^9^,^10^, these materials may be converted in sophisticated and miniaturized devices^11^,^12^. As far as charge conduction is concerned, difficulties involved in achieving good quality sample-electrode contacts hamper the studies of electrical transport properties and magnetoresistance effect in micro/nanowires. The study of the electrical properties and the magnetoresistance effect (MR) in core-shell microwires is important for future applications in the microelectronic field^13^. The giant MR effect has been observed in thin films and colossal effect in doped manganese oxides^14^,^15^. Usually this effect in magnetic coated microwires comes along with outstanding magnetic properties, such as enhanced magnetic softness, magnetic bistability, and giant magneto impedance effect^16^,^17^,^18^. Microwires with semiconducting/magnetic coated shell may have other applications such as conductor micro-cables for telecommunication, miniaturized high voltage transformers, electromagnetic shields, and actuators.

In turn, strategies for assembling hollow micro/nanotubular structures have attracted attention from different areas of research, which includes bioengineering, chemical physics, and material science^19^,^20^. A larger effort to integrate different hierarchically structured oxide semiconductor materials into one device, involving distinct functionalization, has been put forward^21^,^22^. Understanding the effect along with functionalization of hierarchical micro/nanostructured materials can lead to new designs of devices with adapted physical properties to a specific application^23^.

Recently, nanostructures with different morphologies such as nanoparticles, nanobelts, nanorods, and nanowires have been reported^24^,^25^ by using different physical and/or chemical approaches^26^,^27^,^28^. Among them, thermal oxidation process by simply heating a high purity metal is used to produce different micro/nanoscale morphologies^29^,^30^,^31^,^32^,^33^. Interestinlgy, depending on the adopted procedure during the synthesis, nanostructures are formed as a results of completely different mechanisms—by extrusion of metal atoms, lattice, grain, and surface ion diffusion, or by adding oxide molecules on their tips depending on the melting point of each metal and type of architecture. The driving force responsible for each mechanism is thought to be different. It is important to note that although several synthesis methods have been developed, the understanding on the microscopic nature of the growth mechanism along with its driving force are rarely reported in the literature.

Here, we present morphological, structural, and electrical transport studies on metal-semiconductor Zn/ZnO core-shell coaxial microwires. The microwires are comprised of a metallic zinc core and a semiconducting ZnO microshell covered with ZnO nanowires on the top of it. We have studied the MR effect in coaxial core-shell microwire by changing the thickness of the nanostructured shell and compared it to that of the pure metallic microwire. We have found an enhancement of MR in the samples with ZnO nanostructured shell on the surface of the microwires. In addition to scattering surface effects, induced ferromagnetic-like moments in the nanostructured shell can superimpose to the external magnetic field bringing about a larger effective magnetic field in the core.

On the other hand, when the coaxial microcable is produced at higher temperature and longer time, the metallic core evaporates producing hierarchical architectures. We have found out nanowires and nanosticks growning on the outer surface and in the inner part of a microtube, respectively. We suggest that two distinct mechanisms, each one with its driving force, take place simultaneously in order to assemble this interesting multiscale morphology. The simultaneously appearance of nanosticks with nanowires, as far as we know, has not been reported to date. The evolution of the precursor from metal to nanostructured oxides has been accompanied by the change of local electric field gradient, as shown by hyperfine interactions measurements, which revealed the presence of highly textured metallic Zn islands in the interfacial layer between ZnO grains, where nanosticks grow, and ZnO layer where nanowires are formed. The presence of these Zn regions is preserved even after long oxidation times suggesting that the confinement is playing an important role in the growth process. These confined islands are formed of thin flat regions parallel to the surface of the microtube as revealed by electrical field gradient direction. The observation of enclosed regions between interfaces leads to the interpretation of stress-induced ion migration acting as a driving force to grow internal nanosticks. The whole discussion on the formation of hierarchical micro/nanostructured sample compasses different steps involving lattice, grain boundary diffusion, vapor-liquid-solid phases, and stress-induced migration.

## Results

We have grown a semiconducting nanostructured shell on metallic microwires by using the thermal oxidation process in air and above the melting point of Zn. The formation of nanostructures by heating pure metal *via* thermal oxidation is always brought about by the growth of a micro oxide layer on the surface of the metal. The growth mechanism of nanowires occurs by grain boundary, surface, and lattice Zn ion diffusion at high temperature. Usually, temperature is necessary to increase the atomic vibration in the system to overcome energy barriers releasing ionic motion and changing the concentration of the chemical potential. The concentration gradient is often called the driving force of the diffusion process. Interestingly, one can take advantage the fact that depending on the geometry of the starting metal, this thin layer can assume flat, cylindrical, or spherical shapes. Therefore, a self-assemble growth of cylinder, ball or thin film with nanostructures on their surface takes place when metallic precursor of the interested material is heated to high temperature in air.

### Synthesis and *in situ* electric resistivity measurements

**Figure 1:**
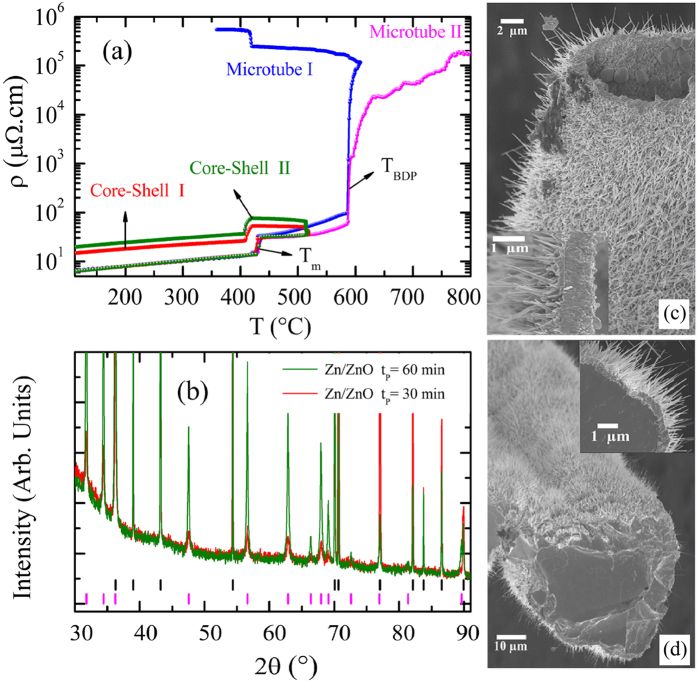
(**a**) Temperature dependence of electrical resistivity obtained during the synthesis of four samples by thermal oxidation of metallic Zn microwires in air. (**b**) X-ray powder diffraction for the two oxidized microwires (30 and 60 min). SEM images show different hierarchical morphologies of oxidized microwire: (**c**) ZnO microtube decorated with nanowires on the surface; (**d**) Zn/ZnO core-shell-type structure.

We have implemented a custom-built dedicated apparatus to perform electrical resistivity measurements simultaneously to the growth of the nanostructures. Figure 1(a) shows curves of electrical resistivity as a function of temperature for four metallic microwires that, as we shall see, turn into different ZnO hierarchically nanostructured morphologies after the thermal oxidation process takes place. It is important to emphasize that, we start with Zn microwires and, depending on the thermal cycle, end up with at least two different morphologies – a coaxial microwire with Zn/ZnO core-shell structure (represented by the so-called core-shell I and core-shell II curves) or a ZnO microtube due to complete evaporation of the metallic core (represented by the so-called microtube I and microtube II curves).

While the electrical resistivity of the microwire is measured, the temperature of the system is raised to 400 °C (just below the melting point of Zn metal). During this window time, a thin layer of ZnO grows on the microwire surface. Next, the temperature of the chamber is raised again to a defined set point, here, T = 515, 600, and 800 °C. Afterwards, the sample is cooled down to room temperature. All curves display a jump around 420 °C revealing the first order phase transition from solid to liquid of Zn metallic microwire. Interestingly, even during the liquid phase, ρ(T) measurement remains uninterrupted. Adhesion and cohesion forces due to wettability of the liquid and the mechanic structural aid provided by the solid thin ZnO layer formed first provide the condition for the measurement^34^.

**Figure 2:**
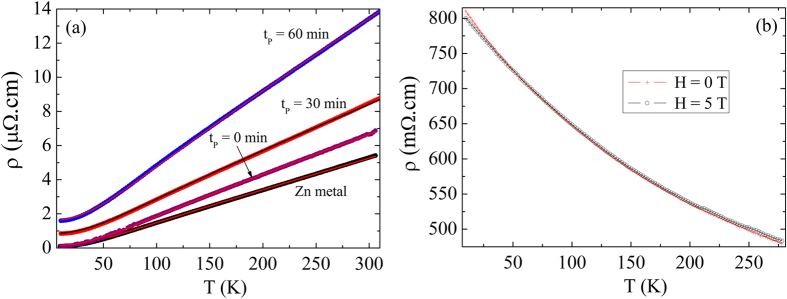
(**a**) Electrical resistivity measurements as a function of temperature along with Bloch-Grüneisen fitting curves for Zn metal microwire and samples produced in different oxidation waiting times tp = 0, 30 and 60 minutes. (**b**) Electrical resistivity measurements as a function of temperature and applied magnetic fields of H = 0 and 5 tesla showing the semiconducting behavior of a completely oxidized microwire.

At 515 °C, during the waiting time of 30 and 60 min at this temperature, the electrical resistivity increases suggesting the formation of an oxide layer on the surface of Zn metal microwire. It is noteworthy that, as the system is cooled down, the sample undergoes the phase transition from liquid to solid revealing reversibility. At the end of the process, the sample is consisted of a metallic core covered with a nanostructured ZnO shell topped by a nanowire array on the surface. Note that there is an increase in its residual electrical resistivity. At room temperature, when the heating process starts we have a metallic microwire with 6.2 μΩ.cm and after the thermal cycle we have an electrical resistivity of 14.2 and 20.0 μΩ.cm for the coaxial Zn/ZnO microwire with exposing time of 30 and 60 minutes, respectively, see also Fig. 1S (Supplemental Materials). Most important, all samples subjected to this heat cycle beyond the liquid phase were found to behave like a metal after the process. In some cases, due to long oxidation time, the sample oxidized completely turning into a semiconductor microwire and an insulating behavior was observed like the so called microtube I and II curves. Figure. 1(c,d) show representative SEM images of samples with different hierarchical morphology obtained with the process indicated by the curves microtube II and core-shell I. The first measurement (core-shell I) corresponds to the fabrication of a coaxial microcable with core-shell morphology where Zn metal is located in the center and ZnO forms an external thin shell covered with nanowires. Figure 1(d) also shows the cross section of a microwire oxidized for t_p_ = 30 min which is composed of Zn metal in the core and a shell of ZnO with nanowires on its surface (ZnO NW). The obtained ZnO NWs are homogeneously dispersed and perpendicularly oriented to the surface of the zinc oxide layer. The ZnO microlayer has an average thickness of 0.6 μm. A large number of ZnO nanowires with direction perpendicular to the surface, length up to 3.0 microns, and average diameter of 90 nm is observed. The sample exposed to an oxidation time of t_p_ = 60 min has a layer of 2.0 μm with larger nanowires. The results of X-ray diffraction measurements are shown in Fig. 1(c) for the two oxidized samples heated for t_p_ = 30 and 60 min at T = 515 °C. It indicated the presence of two different crystallographic phases corresponding to the metallic Zn and oxide ZnO. A detailed analysis involving Rietveld refinement is shown in the Supplemental Materials, see Fig. 2S. A set of Bragg reflections from Zn metallic belongs to the hexagonal structure with *P63/mmc* space group symmetry. The other set from ZnO with hexagonal wurtzite type *P63mc* space group symmetry confirming the formation of structured ZnO. Rietveld refinement reveals the presence of 8% of ZnO and 92% of Zn metal for the sample oxidized for 30 min and 27% of ZnO and 73% of Zn for the microwire oxidized for 60 min. The lattice parameters of all crystallographic phases are also shown in Supplemental Materials.

Most interesting here is the behavior of curve microtube I in Fig. 1(a) where due to the longer waiting time (60 min) at 600 °C, the electrical resistivity jumps abruptly three orders of magnitude. Afterwards, as the system is cooled down, ρ increases rather modestly showing another jump due to reversible liquid-solid phase transition revealing that the sample still has a fraction of metallic regions. As the temperature is lowered, the electrical contacts broke and the measurement stopped at T = 350 °C. We succeed to obtain the microtube, but unfortunately the electrical resistivity was not recorded down to room temperature. A similar jump was observed in curve microtube II where the set point temperature was increased up to 800 °C. Again, the electrical resistivity increases sharply suggesting that the metallic percolation path in the liquid phase breaks down. It also takes place in the curve microtube II. We suggest that the Zn liquid medium loses continuity turning into liquid islands throughout the microwire breaking down the path of electrical current. The presence of this jump in the electrical resistivity suggests that its origin can be associated with the evaporation of metallic Zn islands present along the microwire separated by continuous regions of ZnO. The electrical resistivity of ZnO which may act as a tunnel to charge carriers is much higher than that of Zn metal. We suggest that as time and temperature increase, the metallic islands become smaller increasing the electrical resistivity as displayed in Fig. 1(a). Figure 1(b) shows a SEM image of a hollow ZnO microtube with nanowires on the surface obtained by the complete evaporation of the metal core corresponding to microtube II^35^. Due to higher temperature and longer exposure time, the ZnO NWs grown on the surface of the ZnO microlayer have diameters in the range of 200–300 nm and lengths between 4 and 7 μm. Next, we show results of electrical transport and magnetoresistance effect in the coaxial core-shell microwires produced according to core-shell I and II curves. In the sequence, we study the hierarchical morphologies (microtube with nanowires/nanosticks) produced according to microtube I and II curves, Fig. 1(a).

### Magnetoresistance in coaxial core-shell microwires

Figure 2 shows the temperature dependence of the electrical resistivity at low temperatures from 10 K up to 300 K for pure metallic Zn and Zn/ZnO core-shell microwires obtained by using different waiting times – t_P_ = 0, 30, and 60 min. One can observe a metallic behavior—the electrical resistivity decreases when the temperature is lowered. Interesting, microwires oxidized at longer time resulted to be insulator as shown in Fig. 2(b). In metallic systems, the scattering of charge carriers is caused by the long-range Coulomb potential brought about by the crystal lattice and impurities^36^. The electric resistivity can be expressed as the sum of two contributions *ρ(T)* *=* *ρ*_*0*_ *+* *ρ*_*el-ph*_*(T)*, one is a temperature independent contribution coming from scattering due to impurities and point defects in the crystal lattice; and a temperature dependent part due to interaction with phonons. The Bloch– Grüneisen model has been applied to study the electrical properties of different systems like nanocrystalline metallic films and metallic nanowires^37^,^38^,^39^. In this context, the contribution of electron-phonon interaction to the temperature-dependent electrical resistivity *ρ(T)* is described by the following equation:


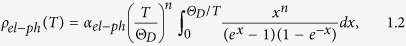


here *α*_*el-ph*_ is the coupling parameter and represent the scattering strength of electron with acoustic phonons, Θ_D_ is the Debye temperature, *T* is the absolute temperature, and *n* is related with the electron interaction nature. The metallic behavior was fitted by the Bloch-Grüneisen equation for all samples as shown in Fig. 2. The fit was performed using three procedures: first, values of *n* and *α*_*el-ph*_ were obtained by fixing Θ_D_ = 327 K. These fits turned out *n* = 2 for all samples and *α*_*el-ph*_ increases monotonically as the waiting time increases. The second procedure consisted in setting *n* = 2 and leaving free Θ_D_ and *α*_*el-ph*_. The results indicated values for the Debye temperature around the theoretical value Θ_D_ ~ 327 K and again *α*_*el-ph*_ increases monotonically. In the last procedure, values of *α*_*el-ph*_ were obtained by fixing Θ_D_ = 327 K and *n* = 2. In all fits, a similar increasing in the coupling parameter was observed with increased oxidation time. The values found by using the last procedure are: ρ_0_ = 0.093, 0.85, 1.6 μΩ.cm and *α*_*el-ph*_ = 9.89(6) × 10^−6^, 8.77(4) × 10^−6^, and 1.37(9) × 10^−5^ Ω.cm for t_P_ = 0, 30 and 60 min, respectively.

The results show that both the electron-phonon coupling and the residual electrical resistivity increase as the oxidation time increases. The electric resistivity magnitude at room temperature of nanostructured-ZnO samples is also higher when compared to the Zn metal. The different geometric factor associated with the reduction of the cross section was taken into account. This result may also be associated with oxygen diffusion in the metallic core at high temperature. However, the solubility of oxygen in liquid Zn metal is very low—for example, at T = 550 °C, the amount of diluted oxygen is 3.7 × 10^−5^ at% (atomic percentage)^40^. Energy dispersion spectroscopy has revealed the presence of ~ 6% of oxygen in a region very close to ZnO layer while in the center of the core the amount is negligible for the sample with t_p_ = 60 min (not shown). Turning back to x-ray diffraction results, we can see a splitting of Bragg reflection peaks of the metallic phase (see supplemental materials, Fig. 3S) in the heat treated sample. This result indicates the presence of two metallic hexagonal structures with slightly different lattice parameters in the core, which may be the source of higher charge carrier scattering.

**Figure 3:**
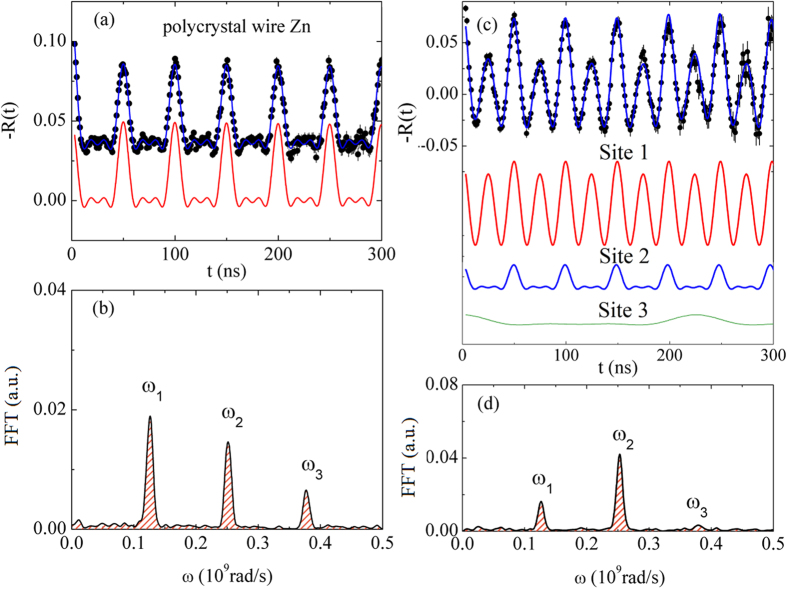
Spin rotation R(t) spectra at room temperature with their corresponding fast Fourier transform of the spin precession frequencies ω_n_ for pure metal before (**a,b**) and after oxidation process (**c,d**).

In order to obtain a better understand on the microscopic structure and its consequence in the electrical conductivity, we have carried out a local investigation by using PAC spectroscopy in the Zn metallic microwire covered with ZnO nanostructures. As described in the experimental procedure, after the diffusion of the probe nuclei into the metallic microwire, PAC measurements were performed in pure Zn metal wire at room temperature. After this measurement, the same sample was heat treated in air to perform a thermal oxidation process to produce a core/shell structure similar to core-shell I curve. Figure 3 shows the spin rotation spectra -R(t) with corresponding fast Fourier transform (FFT) of the spin precession frequencies ω_n_ measurements for both pure Zn metal and oxidized samples. The analysis of the data has been done taking into account only quadrupole interactions. Results, displayed in Fig. 3(a), show a well-defined single frequency, corresponding to ^111^In(^111^Cd) at substitutional sites of Zn metal hexagonal structure, with ν_Q_ =  133.9(2) MHz, which is in very good agreement with values found in the literature for ^111^Cd at Zn sites in polycrystalline Zn structure^41^. It is important to mention that for single crystal samples the values of frequencies are not different from those of polycrystalline ones. However, the amplitude coefficients *S*_2n_ of frequencies ω_n_ are different depending on the orientation of EFG with respect to the plane that contains the detectors in the experimental apparatus.

**Figure 4:**
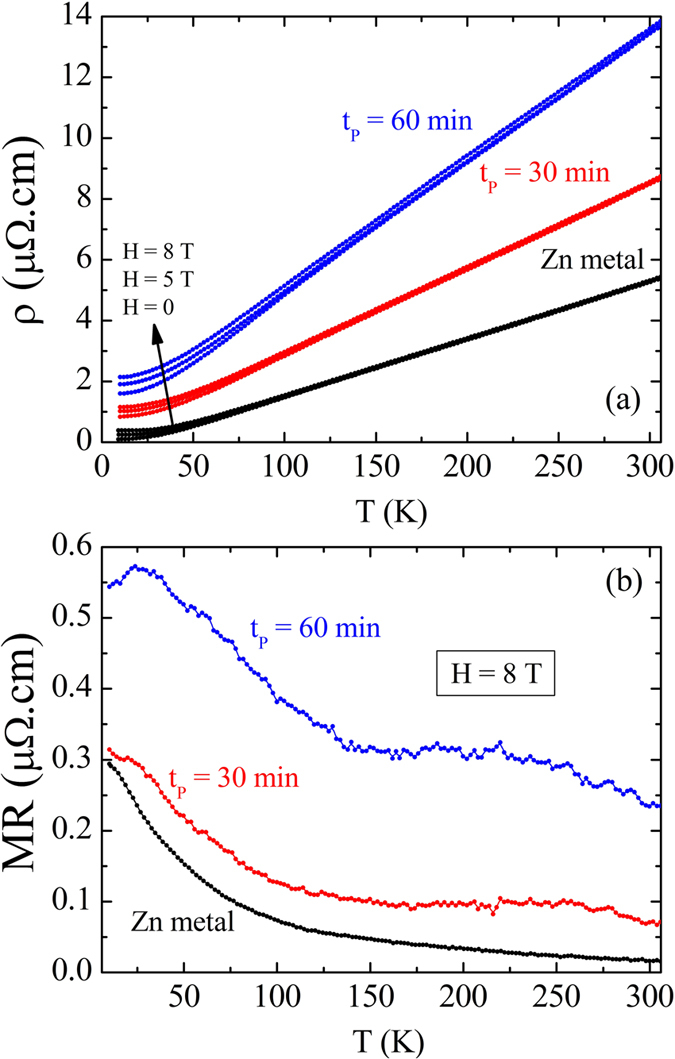
(**a**) Electrical resistivity as a function of temperature and in the presence of magnetic field H = 0, 5, and 8 T for a pure metallic microwire and two core/shell coaxial microwires heated at different waiting time. (**b**) Magnetoresistance effect measured at magnetic field of H = 8 T as a function of temperature.

The obtained spin rotation spectrum of the same sample, but after thermal oxidation (see Fig. 3c), is more complex than that for pure metal. This PAC spectrum was fitted by considering three different site positions for ^111^Cd probe nuclei—each one showing a distinct well-defined frequency and axial symmetry (η = 0). The first site has the highest population with a volume fraction of *f*_1_ = 50% and quadrupole frequencies of 133.9(2) MHz. The site 2 with fraction, *f*_2_ = 35%, has similar quadrupole frequencies of 132.7(1) MHz. Both sites belong to metallic Zn structure. However, as one can see in Fig. 3(c), the functions representing each fraction are very different. The function that represents site 1 corresponds to a situation where the EFG of each nuclear probe is all oriented along the same direction. This is typically found in single crystals or highly textured structures^42^. Site 2 is represented by a function similar to that displayed in Fig. 4(a) which corresponds to probe nuclei replacing Zn ions in a polycrystalline environment. Finally, site 3, with lowest population (*f*_3_ = 15%) corresponds to probe nuclei at Zn sites in ZnO hexagonal structure, with ν_Q_ = 31,0(2) MHz^43^,^44^. These results are in agreement with XRD suggesting the existence of two metallic phases with different lattice parameters and highly textured. The textured phase would be at the interface induced by ZnO crystal lattice and the other in the core. The applied electrical current induces a magnetic field, which in turn creates a radial force pushing the charge carriers to the surface. Therefore, the electrical current has a tendency to go through the surface of the metallic core where both phases (Zn and ZnO) are connected and oxygen diffused causing a strong scattering of charge carriers.

Figure 4(a) shows the electrical resistivity as a function of temperature and in the presence of magnetic fields of H = 0, 5, and 8 T for the pure Zn metal microwire and two other microwires with core-Zn/shell-ZnO coaxial type structure. As one can see the pure Zn sample present a positive magnetoresistance effect (enhancement of electrical resistivity) most pronounced at low temperatures. In a pure metal, the application of a magnetic field deflects the free charge carriers deviating from the direction of the electric current and increasing the scattering, which in turn increases the electrical resistivity. We have observed a similar behavior in the core-shell coaxial microwires, but the effect is much higher. In order to quantify this effect, we have defined MR = ρ(T, H) – ρ(T, H = 0)], where ρ(T, H) and ρ(T, H = 0) is the electrical resistivity measured with and without magnetic field, respectively. Before applying this definition, we have subtracted out the residual electrical resistivity taken at the lowest measured temperature and H = 0 from each curve. Interestingly, as shown in Fig. 4(b), the MR effect is observed in the whole temperature range, but much more pronounced at low temperature for all samples.

### Microtube with formation of nanosticks in its inner surface

**Figure 5:**
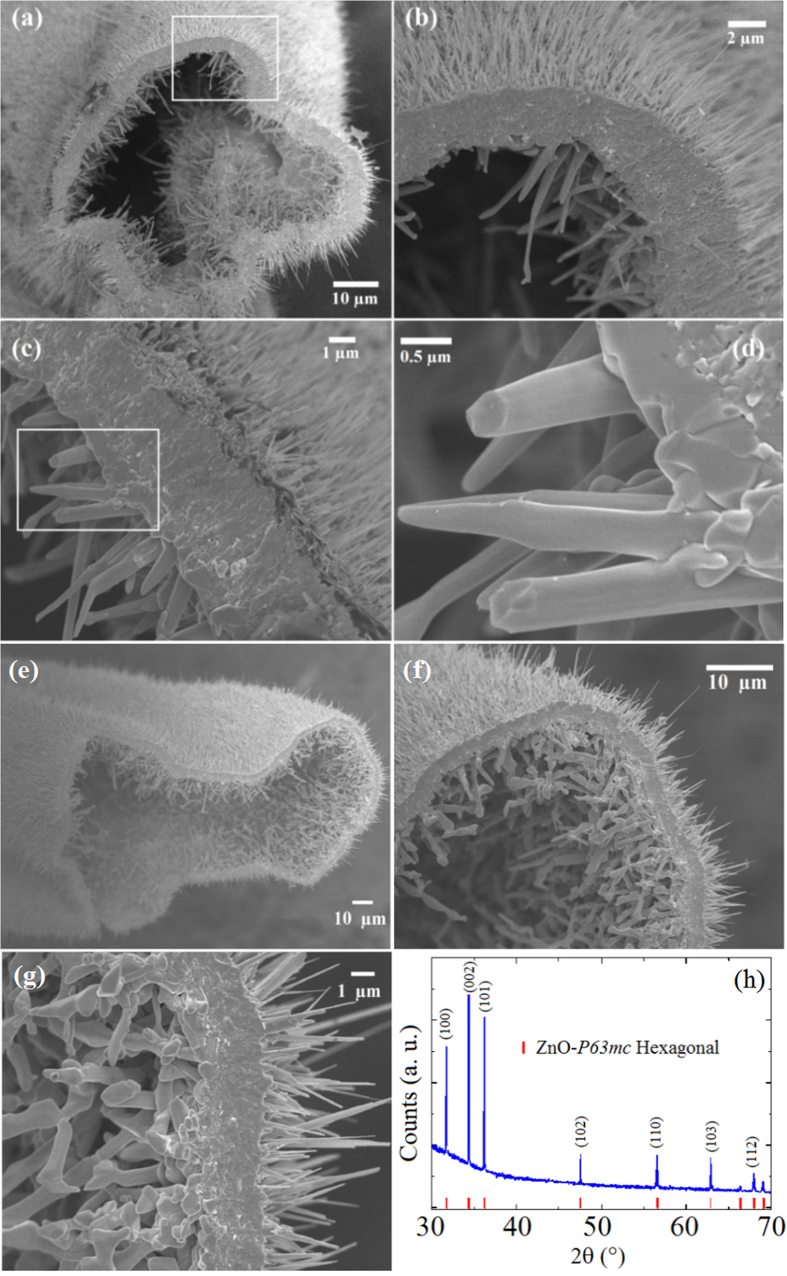
(**a–d**) Sequence of SEM images of a microtube decorated with ZnO nanosticks (inner part) and nanowires (outer surface). (**e**–**g**) SEM images of a larger ZnO microtube and (**h**) X-ray diffraction measurement indicating a single-crystalline phase belonging to ZnO *P63mc* space group symmetry.

When the coaxial microcables are produced at higher temperature and longer exposure time, the metallic core may partially or completely evaporate turning into hierarchical architectures as represented by the curves microtube I and II in Fig. 1(a). We have found nanosticks growing on the inner surface of microtubes decorated with nanowires at the outer surface, as shown in Fig. 5(a–g).

The obtained samples were cut in several segments after the oxidation process and depending on the thermal cycle, some sections presented hollow tube and others a thin metallic layer in the inner part of the tube. Most important here was the observation of spontaneous growth of discrete nanosticklike structures on the inner surface of the microtube. SEM images of such microtubes are displayed in Fig. 5. The nanosticks have different morphology when compared to nanowires on the surface and have diameters from 290 to 650 nm and lengths in the range of 1.4 to 3.5 μm. The difference in the nature of the precursor and the presence of solid, liquid, and vapor phases strongly suggest that the growing mechanism is different from that of the external nanowires. Figure 5(e–g) shows another hierarchically structured microtube with larger diameter revealing the reproducibility. We believe that with further improvement on the processing parameters a more regular shape of the microtube and nanosticks can be obtained. Additionally, the SEM images of this second microtube show a higher density of nanostructures in both internal and external surfaces. The resulting XRD pattern for this sample reveals a single-crystalline phase of ZnO with the same *P63mc* space group symmetry of nanowires. As far as this point is concerned, we have studied the evolution of the Zn metallic precursor from a local point of view in order to shed light on the nature of the mechanism responsible for the growth of nanosticks inside the microtubes.

**Figure 6:**
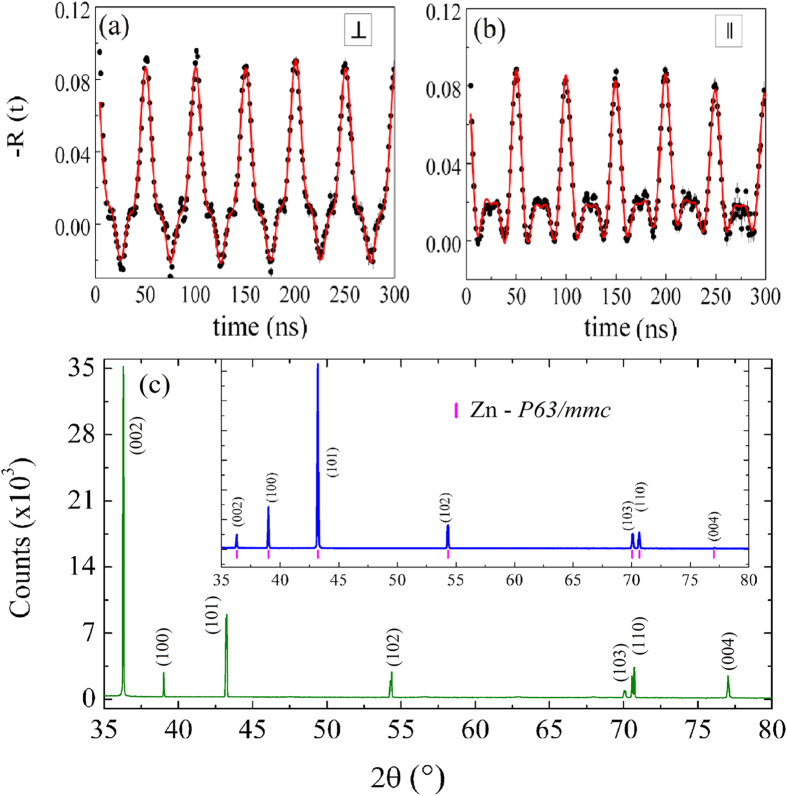
R(t) spectra at room temperature for: (**a**) perpendicular and (**b**) parallel configuration relative to the plane of detectors for a hollow microtube obtained at 800 °C for 5 hours. (**c**) XRD pattern of the oxidized Zn microwire for 30 min at 515 °C revealing preferred orientation of (002) plane of the metallic phase. The inset shows XRD of a pure isotropic policrystalline metallic Zn microwire.

We have carried out a local nuclear investigation in the Zn metal precursor while it turns into nanostructured ZnO. As we increase the temperature and exposing time, the metallic phase is consumed and both nanostructures, at the inner and outer surfaces of the microtube, coalesce into a continuous layer and disappear. Figure 6(a,b) shows spectroscopy nuclear measurements in samples heat treated at T = 800 °C with an annealing time up to 5 h. Unexpectedly, the presence of metallic phase as islands confined at the interface of oxide layers was observed. XRD indicates only ZnO crystal phase even in the sample heat treated at lower temperature and time as shown in Fig. 5(h). In order to check anisotropic nature, PAC measurements were carried out taking into account two different configurations. The longitudinal axis of the microtube was oriented in a perpendicular and parallel direction relative to the plane of detectors as showed in Fig. 6(a,b). First, both spectra belong to long ranged metallic structure of Zn revealing its presence. Second, the resulting R(t) along with its corresponding *S*_*n*_ reveal a strong dependence of the electric field gradient direction with the plane of detector for these confined islands. The PAC spectra now were fitted by considering two sites. Site 1 with major population was modeled by a function that represents fully textured metallic Zn with ν_Q_ = 133.9(2) MHz and site 2 presented a ZnO characteristic quadrupole frequency ν_Q_ = 31.0(2) MHz. Surprisingly, the metallic Zn forms thin flat highly textured regions (islands) parallel to the surface of the tubes or probably as single crystal confined between ZnO layers. In other words, highly textured metallic Zn is formed as thin flat regions parallel to the inner surface of the microtube.

Turning back to XRD measurements, one can see the preferred orientation of a crystallographic plane. Figure 6(c) shows XRD for the sample heat treated for 30 min. The ZnO phase has low intensity, but can be seen in an enlarged scale, Fig. 1 (c). The inset of Fig. 6(c) displays the XRD for the policrystal pure metallic microwire. One can see that the plane (002) is supposed to be very low for polycrystalline Zn phase, but it has a very high intensity for the heat treated sample revealing preferred orientation. These combined results indicate spontaneously orientation of (002) plane at T = 515 °C and the existence of them as a strongly oriented crystalline domains closely resembling single crystals at higher temperature.

While PAC characterizations reveal the presence of textured Zn metal in the hierarchically (nanosticks and nanowires) microtube, X-ray diffraction pattern indicates single phase ZnO structure probably due to the existence of two protecting thick ZnO layers. Our experiment revealed that the ^111^In (^111^Cd) probes have a preference to occupy metallic Zn sites. This result shows a strong potential of PAC spectroscopy to disclose confined nanostructured regions and their orientation in the microtube matrix. We will argue that the evolution of these confined metallic regions play an important role to set the stage for the growing of nanostructures. It is reasonable to consider that any mechanism of ZnO growth involves these highly oriented confined metallic regions as a source of Zn ions for diffusion. In the discussion section, we put an effort to rationalize the growth mechanism focusing on the driving forces responsible for the formation of the nanostructures inside the microtubes.

## Discussion

We have produced coaxial microwires comprised of a metallic zinc core and a semiconducting ZnO microshell covered with ZnO nanowires on the top of it. Interestingly, as shown in Fig. 4(c), the MR effect is observed in the whole studied temperature range, but much more pronounced at low temperature for all samples. This high value of MR effect may come from contributions of induced magnetic field in the nanostructured ZnO shell. Bulk ZnO is a diamagnetic material due to completely filled *d*-shell – the effective magnetic moment is zero. When one applies a magnetic field on a diamagnetic material, it creates an induced magnetic field in an opposite direction. Therefore, if the ZnO covered shell were diamagnetic, it would produce a shielding-like effect to the external magnetic field. On the other hand, ferromagnetic-like behavior in nanostructured samples of ZnO has been observed^8^,^45^,^46^. It has been shown that all metal oxides in nanostructured form reveal ferromagnetic-like properties which is absent in bulk samples^47^,^48^,^49^,^50^. The idea is that the origin of ferromagnetism in these materials is due to the appearance of localized electron spin moments resulting from uncompensated charges and/or intrinsic defect at the grain boundary. In the present case, during the oxidation of the Zn microwires, cationic mobility takes place throughout the lattice. The diffusion of Zn coming from the liquid core is necessary to grow the nanowires on the top of the microlayer. During this process Zn intersticial sites and oxygen defects are formed in the crystal lattice. Within this context, the charge carriers inside the metallic core may experience an effective magnetic field that is the sum of the external magnetic field and the magnetic field induced by magnetic moments originated from defects. On the other hand, the presence of two metallic phases creating scattering interfaces may also contribute to enhancement of MR effect.

As the temperature and oxidation time increase, we have observed the formation of hierarchically structured nanowires on the microtube with nanosticks in its inner surface. We suggest that both migration of ions and vapor-solid processes play a role in growing micro/nanostructures and transforming metal microwires into hierarchical nanostructured microtubes. At temperatures above the Zn melting point, the vapor pressure of Zn increases and a significant amount of Zn would evaporate into the atmosphere leaving a hollow microtube with a thin Zn liquid layer covering its inner surface. On the other hand, the oxygen in the atmosphere will enter inside the microtube. The oxygen can migrate directly to the liquid layer forming solid ZnO grains or reacting with Zn vapor released from the core to form ZnO in gas phase. Interesting, the solubility of oxygen in liquid Zn metal can be neglected—for example, at T = 700 °C, the amount of diluted oxygen is 9.3 × 10^−4^ at% (atomic percentage)^43^. At this temperature range, vapor-solid process contributes for the growing of grains in the inner part, rather than solely lattice/grain/surface ion diffusion on the outer shell of the tube. As the process goes on some ZnO nucleated grains eventually coalesce resulting in larger regions. Intuitively, these processes would result in a continuous ZnO ticker layer inside the microtube. At first glance, nanosticks grow from the root of large individual grains. Another possibility would be condensation of ZnO gas on the nucleation sites such as grains and nanostick tips. Why this scenario would lead to the formation of discrete nanosticks instead of a continuous layer is unknown.

**Figure 7:**
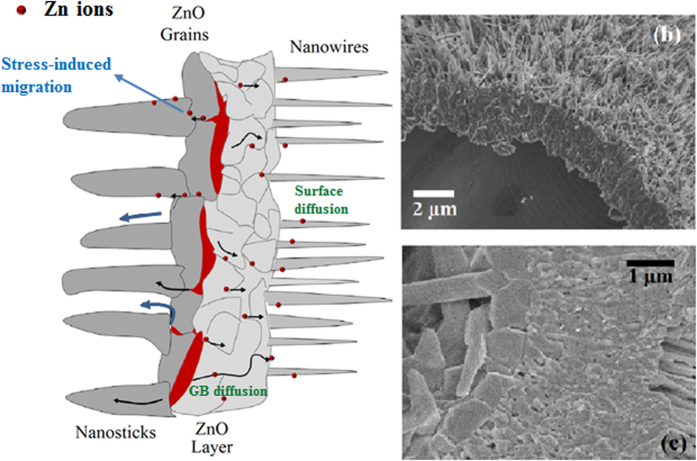
(**a**) (This figure was drawn by the first author Cynthia M. Rivaldo-Gómez) Sketch of different regions observed in the ZnO microtubes illustrating the growth mechanism of the internal and external nanostructures of the microtubes. The metallic regions in red between layers are detected by PAC. (**b**) SEM image of a microtube in an intermediate stage showing a thin layer of Zn metal covering the inner surface. (**c**) Transversal section of ZnO microtube showing the presence of a chain of ZnO grains and a continuous ZnO layer.

Figure 7 shows a sketch of the whole growing process of hierarchically micro-nanostructured microtube along with supporting SEM images. The formation of nanosticks and nanowires in the inner part and on the surface of the ZnO microtube is closely related to Zn ions migration in opposite directions due to different driving forces. First, a thin zinc oxide layer is formed due to reaction with oxygen in the atmosphere preserving the shape of the microwire. In a second stage, above the melting point, the nucleation and growth of nanowires are formed as a result of grain boundary, lattice and surface Zn ions diffusion from the liquid inner part across the thin ZnO layer due to chemical potential gradient. This mechanism describing the growth of NWs is governed by Arrhenius law^51^,^52^. During this stage, the ZnO solid layer covered with nanowires encloses the Zn metallic liquid which partially evaporates leaving a thin Zn layer as can be seen in Fig. 7(b). At this stage, ZnO grains start to be formed as a product of reaction between the vapor and liquid Zn with oxygen molecules, present in the atmosphere. The formation of a layer comprised of grains encloses metallic islands at the interface between ZnO grains and ZnO layer. The confirmation by PAC measurements of the presence of high textured Zn metal leads to the suggestion of stress-induced migration mechanism for the nanosticks growth. To explain the growth of nanosticks we may consider two thermodynamic points: (1) at high temperatures near the Zn boiling point, the vapor pressure increases causing a stress between metallic liquid islands and both ZnO solid interfaces and (2) the mismatch between oxide layers and liquid metal due to different thermal expansion coefficient. Following these considerations, we may suggest that the presence of a hydrostatic gradient pressure and the compressive stress generated in the metallic regions at interfaces induces the radially inward migration of Zn ions. The driving force for the ionic diffusion in the direction from more to less compressive regions is described by Fick´s law^53^ and is usually found in multilayer thin films^54^,^55^.

## Conclusion

Summarizing, ZnO microtubes with growth of nanosticks inside and nanowires on the outer surface as hierarchical micro/nanoarchitecture have been reported. First, a coaxial core/shell microwires is obtained—the core is formed by metallic Zn and the semiconducting shell is comprised by a thin oxide layer covered with a high density of nanowires. We have found an enhancement of MR effect in the samples with ZnO nanostructured shell on the surface of the microwires. We believe that induced ferromagnetic-like moments in the nanostructured shell and nanowires superimposes to the external magnetic field. This effect brings about a larger effective magnetic field in the core, which in turn enhances the MR effect in the hierarchically nanostructured samples. Second, microtubes, formed due to complete evaporation of the core, decorated with nanowires on the external surface are obtained. In an intermediate synthesis stage, between coaxial core/shell structure and microtubes formation, a hierarchical morphology comprised of nanosticks in the internal surface of the tube has been discovered. The synthesis process of this hierarchical micro-nanostructured material, going beyond the melting point of Zn and the evaporation of the metal, was accompanied by *in situ* electrical resistivity measurements. PAC spectroscopy measurements revealed the presence of highly textured metallic Zn in the interfacial layer between Zn polycrystalline metal (core) and the thin layer of ZnO. Hyperfine interactions measurements showed the presence of confined metallic Zn regions, even after long oxidation times. The x-ray diffraction indicates growth in the *c*-direction, (002) plane. Unpredictably, the metallic Zn clusters form highly textured thin flat regions oriented parallel to the surface of the microtube. The driving force to boost the growth of internal nanosticks has been attributed to stress-induced migration of Zn ions due to compressive stress caused by the presence of confined regions at the interface between ZnO layer and chain of ZnO grains.

## Methods

### Synthesis and *in situ* electrical resistivity measurements

The synthesis of ZnO nanostructured shell on metallic microwires (50 μm) was carried out in a quartz tube furnace by using the thermal oxidation process. While the oxidation process of the Zn microwire takes place, electrical resistivity was measured *in situ* with a four-probe method by using a custom-built dedicated apparatus. Measurements of the electrical transport properties of free microwires are very rare due to the difficulty involved to obtain good quality microwire-electrode contacts. Measurements in smaller systems, such as nanowires, are usually done by encapsulating the sample in a template or in a substrate and the electrical resistivity is taken by two-contact method. Here, silver epoxy was used to connect the Zn microwire-electrodes with the four-probe station – the station is composed of alumina and the wiring is made from platinum. Therefore, the microwire is perpendicularly placed on the four parallel platinum wires which are fixed on a platform made of alumina. It is worth noticing that the microwire is held on top of the four parallel probes, not lying down in any kind of surface. Data was collected in both air and gas flow by heating the system up to a temperature above the Zn melting point and followed by a cooling stage down to room temperature.

### Sample Characterizations

X-ray diffraction (XRD) was carried out in a diffractometer STADI-P model Store and scanning electronic microscopy (SEM) images were obtained using a JEOL JMS-6701F FEG-SEM. At low temperatures, electrical resistivity measurements as a function of temperature and magnetic field were performed by using the Physical Property Measurement System from Quantum Design.

### Perturbed angular correlation (PAC)

PAC spectroscopy was carried out using ^111^Cd probe nuclei produced from the electron-capture decay of the radioactive ^111^In^+3^. The probe is incorporated into the metallic Zn microwires by thermal diffusion as follow. A volume containing approximately 100 μCi of ^111^InCl_3_ solution was dropped on the surface of the microwires, which were encapsulated into quartz tubes under low pressure of helium (He) atmosphere and heated at 370 °C for several hours. The PAC method is based on the observation of hyperfine interactions between extranuclear magnetic hyperfine field (B_hf_) and electric field gradient (EFG) tensor with nuclear moments at the intermediate level of a cascade decay with the emission of two successive gamma photons (γ_1_ and γ_2_). This technique measures the time evolution of the γ_2_ emission pattern, which is affected by the hyperfine interactions, in coincidence with the γ_1_ detection. The resulting spin rotation spectrum, *R*(t), is modeled by the expression: *R*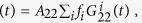 where *f*_*i*_ is the relative fraction of given populated site, *A*_*22*_ is the anisotropy parameter of the probe nuclei and 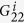 are the corresponding perturbation factors. For the nuclear spin *I* *=* *5/2*, one can use the expression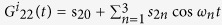, where *ω*_*n*_ are the frequencies of spin precession and *s*_*n*_ are the amplitude coefficients that depend on the single or polycrystalline form of the sample. The spin precession frequency 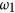 is related with the quadrupole coupling constant frequency (ν_Q_) by the following relation: 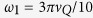, with 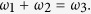 On the other hand, ν_Q_ is derived by the expression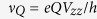, where *Q* is the nuclear electric quadrupole moment of the intermediate level and *V*_*ZZ*_ is the largest component of the EFG tensor. For the study of non-axial symmetric interactions, the asymmetry parameter is obtained by the relation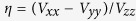, where *V*_*kk*_ (k = x, y, z) denote the components of EFG tensor. In the case of high symmetry interactions η is zero. The values of ν_Q_ and η are determined taking into account that the electric quadrupole moment of the intermediate level of ^111^Cd is known^56^. More details about the PAC technique and experimental procedures can be found elsewhere^57^. After introducing the nuclear probe into the sample, thermal oxidation process was performed in air followed by PAC measurements in order to systematically follow the formation of nanostructures from local structure changes of the metallic microwire precursor.

## Additional Information

**How to cite this article**: Rivaldo-Gómez, C. M. *et al*. Hierarchically structured nanowires on and nanosticks in ZnO microtubes. *Sci. Rep*. **5**, 15128; doi: 10.1038/srep15128 (2015).

## Supplementary Material

Supplementary Information
