# Supplementary material for: Hierarchically structured nanowires on and nanosticks in ZnO microtubes
Source: Sci Rep. 2015 Oct 12;5:15128. doi: 10.1038/srep15128 (PMC4601030; doi:10.1038/srep15128)
Supplement: Supplementary Information [file srep15128-s1.doc]

**Hierarchically structured nanowires on and nanosticks in ZnO microtubes**

C. M. Rivaldo-Gómez^1^, G. A. Cabrera-Pasca^1^, A. Zúñiga^1^, A. W. Carbonari^2^, and J. A. Souza^1^

^1^ Universidade Federal do ABC, Santo André – São Paulo 09210-580, Brazil

^2^ Instituto de Pesquisas Energéticas e Nucleares, Universidade de São Paulo, 05508-000 São Paulo, Brazil


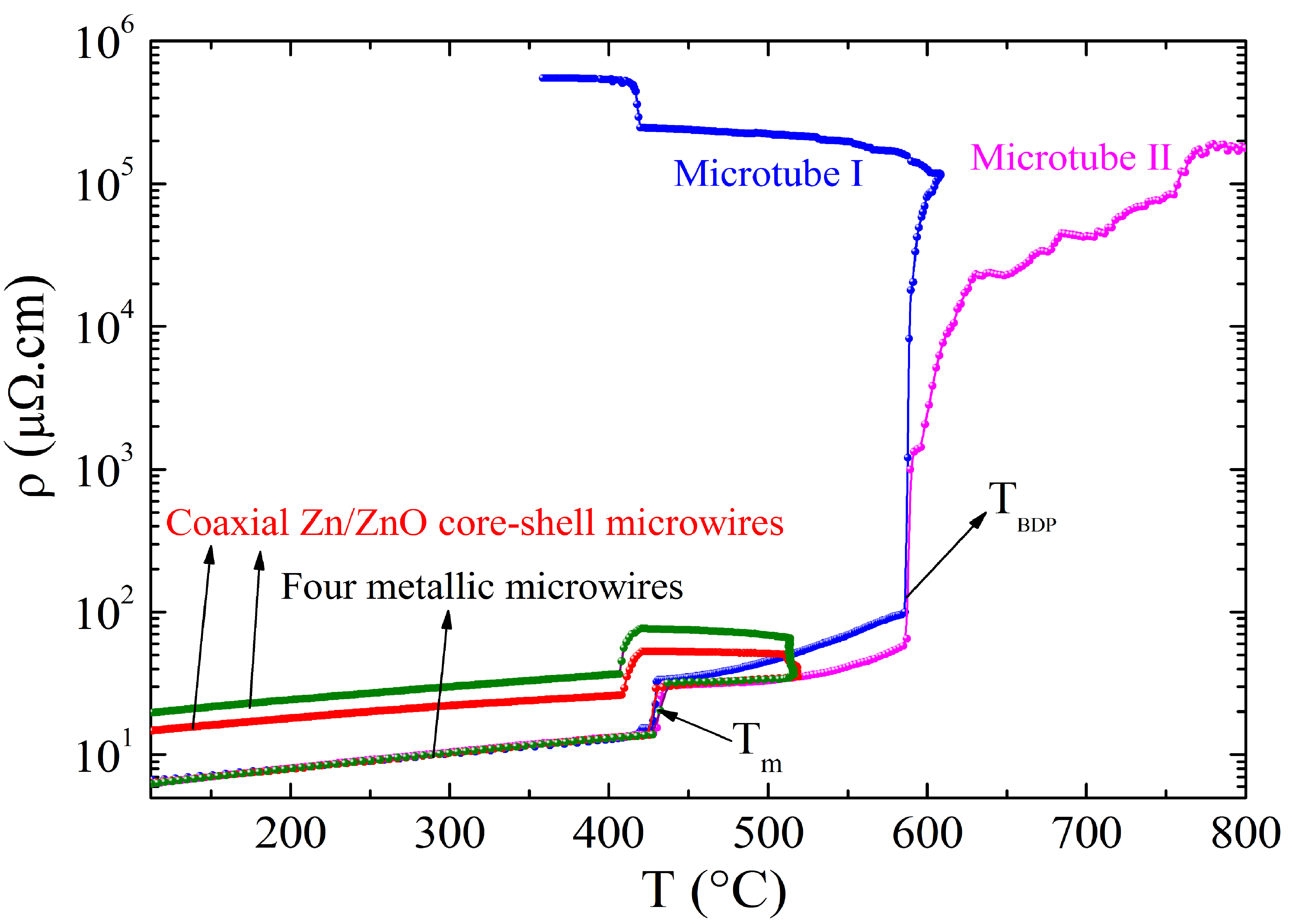


Figure 1S. Temperature dependence of electrical resistivity obtained during the synthesis of four samples by thermal oxidation of metallic Zn microwires in air. The process starts from room temperature measuring the metallic microwire up to melting point. Above the melting point diffusion processes are accelerated and Zn/ZnO core-shell microwire is formed. Increasing the temperature and time, microtubes can also be produced.

We have performed Rietveld refinement on our three samples: Zn metallic microwire, Zn microwire oxidized for 30 and 60 minutes at T = 515 C. The x-ray diffraction pattern along with Rietveld refinement are shown in Fig 2S. In Fig. 2S(a), we show the Rietveld refinement for the as received metallic microwire. Zn metal crystallizes in the hexagonal structure with *P63/mmc* space group symmetry; the obtained lattice parameters are *a* = *b* =2.6645(1) Å and *c* = 4.9463(1) Å. In Fig. 2S(b), we show the analysis for the metallic microwire heat treated for 30 minutes at T = 515 ºC. It is very interesting that we observe two metallic phases with slightly different lattice parameters (see Fig. 3S). The first phase, Zn1, has *a* = *b* = 2.6625(1) Å and c = 4.9454(2) Å while the second Zn2 has lattice parameters *a* = *b* = 2.6667(1) Å and c = 4.9469(3) Å. It is important to mention that the intensity of the Bragg plane (002) is supposed to be very low for polycrystalline Zn metallic phase (see Fig. 2S(a)), but it has a very high intensity (see Fig. 2S(b)) for the heat treated sample revealing preferred orientation. Besides the two metallic phases, we have observed the growth of ZnO on the surface of the microwire with hexagonal structure and *P63mc* space group symmetry. The lattice parameters are *a* = *b* = 3.2488(4) Å and *c* = 5.206(1) Å. The refinement reveals 8% of ZnO phase and 92 % of Zn metallic. In Fig. 2S(c), we show the analysis for the metallic microwire heat treated for 60 minutes at T = 515 ºC. We have observed two phases: a metallic with lattice parameters *a* = *b* = 2.6641(1) Å and *c* = 4.9458(1) Å and ZnO with *a* = *b* = 3.2496(1) Å and *c* = 5.2054(3) Å. The refinement shows that the fraction of ZnO crystal phase has increased to 27 % and Zn metallic decreased to 73 %.


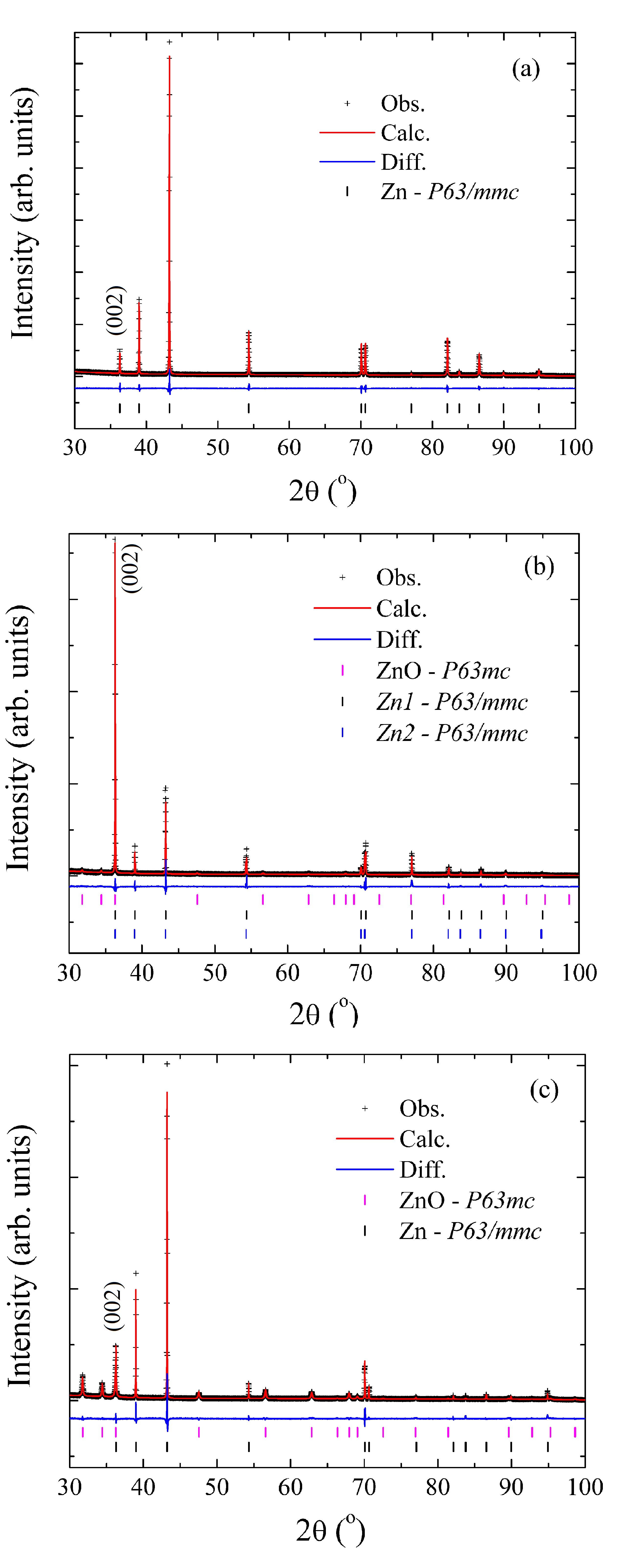


Figure 2S. The x-ray diffraction pattern along with Rietveld refinements for (a) Zn microwire, Zn microwire oxidized for 30 (b) and 60 (c) minutes.


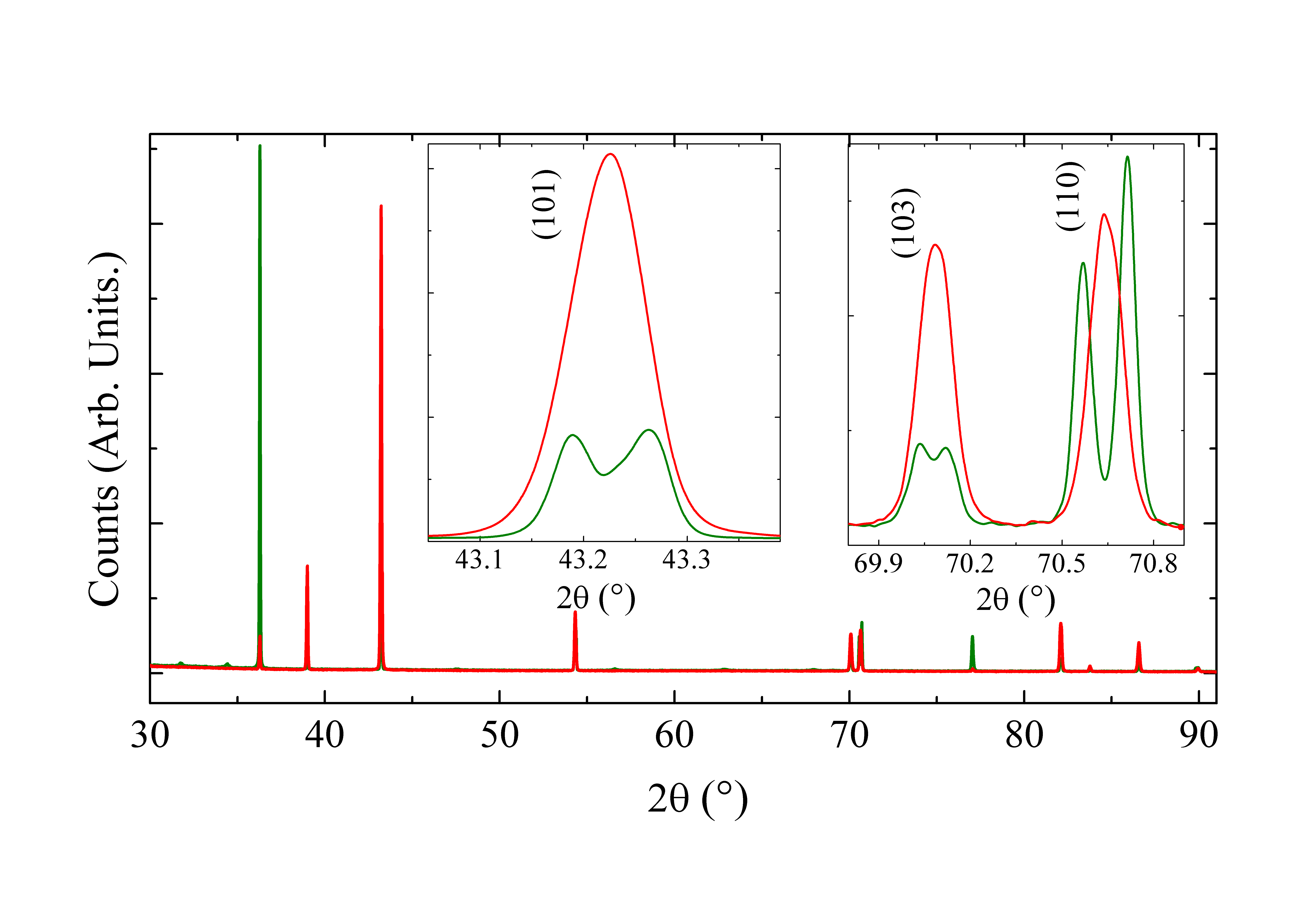


Figure 3S. X-ray diffraction of both pure metallic microwire (green) and other heat treated for 30 min (red). The insets show the planes (101), (103), and (110) of the metallic phase. The mentioned reflections for pure microwire are single while for the heat treated sample above the melting point it turns into a doublet suggesting two metallic phase with slightly different lattice parameters.
